# Genetic polymorphism of interleukins 6 and 17 correlated with apical periodontitis: A Cross-sectional study

**DOI:** 10.1590/0103-6440202305486

**Published:** 2023-12-22

**Authors:** Rebeka Thiara Nascimento dos Santos, Luísa Priscilla Oliveira de Lima, Maria Tereza Cartaxo Muniz, Pâmella Recco Álvares, Márcia Maria Fonseca da Silveira, Ana Paula Veras Sobral

**Affiliations:** 1 Departament of Stomatology and Oral and Maxilofacial Patology, University of Pernambuco, Recife, Pernambuco, Brazil.; 2 Department of Molecular Biology, University of Pernambuco, Recife, Pernambuco, Brazil.

**Keywords:** genetic polymorphism, interleukin-6, interleukin-17, periapical periodontitis, periapical abscess

## Abstract

Interleukins 6 and 17 act in bone resorption in the presence of infections of endodontic origin for host defense. Genetic polymorphisms may be associated with increased bone loss, represented by areas of large periapical lesions. This study aimed to verify the frequency of interleukin 6 and 17 gene polymorphism in patients with asymptomatic apical periodontitis or chronic apical abscess and to verify the existence of correlations between periapical lesion area with age, gender, and presence of the polymorphism, in the studied population, in the state of Pernambuco. A population consisting of thirty diagnosed individuals was included. The area of the lesions was measured in mm². Genomic DNA was extracted and genotyping was performed by Polymerase Chain Reaction Restriction Fragment Length Polymorphism for interleukin 6 (rs 1800795) and interleukin 17 (rs 2275913). Fisher's exact, chi-square, and odds ratio tests were used. A logistic regression analysis was also performed using sex, age, and the presence of polymorphism as covariates, in addition to linear regression to test the relationship between age and lesion area. All tests used a significance level of 0.05% (p ≤0.05%). There was no statistical significance in the occurrence of large areas of periapical lesions correlated with age, sex, and diagnosis, nor in the distribution of alleles in the polymorphism of interleukins 6 and 17 in the studied groups. The frequency of homozygous and heterozygous polymorphism was high. The polymorphism of these interleukins is not correlated with the increase in the areas of asymptomatic periapical inflammatory lesions.

## Resumo

As interleucinas 6 e 17 atuam na reabsorção óssea na presença de infecções de oriegem endodôntica para defesa do hospedeiro. Polimorfismos genéticos podem estar associados ao aumento da perda óssea, representada por áreas de lesões periapicais grandes. O objetivo deste estudo foi verificar a frequência do polimorfismo dos genes interleucina 6 e 17 em pacientes com periodontite apical assintomática ou abscesso apical crônico e verificar a existência de correlações entre área de lesão periapical com idade, sexo e presença do polimorfismo, na população estudada, no estado de Pernambuco. Foi incluída uma população constituída por trinta indivíduos diagnosticados. A áreas da lesões foram medidas em mm². O DNA genômico foi extraído e a genotipagem realizada por Polimorfismo de Comprimento de Fragmento de Restrição de Reação em Cadeia da Polimerase para interleucina 6 (rs 1800795) e interleucina 17 (rs 2275913). Os testes exato de Fisher, qui-quadrado e odds ratio foram utilizados. Uma análise de regressão logística também foi realizada usando sexo, idade e presença de polimorfismo como covariável, além de regressão linear para testar a relação da idade e área da lesão. Todos os testes utilizaram um nível de significância de 0,05% (p ≤0.05%). Não houve significância estatística na ocorrência das áreas grandes de lesões periapicais correlacionadas com idade, sexo e diagnóstico nem nas distribuições de alelos no polimorfismo das interleucinas 6 e 17 nos grupos estudados. A frequência de polimorfismo homozigoto e heterozigoto foi alta. O polimorfismo dessas interleucinas não está correlacionado ao aumento das áreas das lesões inflamatórias periapicais assintomáticas.

## Introduction

Apical periodontitis (AP) is a set of immunological reactions in the periapical tissues, mediated by the activation of the immune system, leading to the recruitment of cells and the production of specific mediators, preventing the spread of infection to other sites in the body. ^1^. Interleukins (ILs) constitute a group of cytokines that act as messengers and are one of the main molecules that act in the immune system, allowing the communication of signals between cells, and coordinating biological processes, with pro or anti-inflammatory stimuli ^2^. 

The production of interleukins varies widely between individuals. These differences can be explained by the presence of genetic polymorphism or SNPs (single nucleotide polymorphism) ^3^, conceptualized as the most common variations in the deoxyribonucleic acid (DNA) chain itself, in a frequency greater than 1% of the population ^4^. SNPs are biallelic and occur in any different combinations between the nucleotides Adenine (A), Thymine (T), Cytosine (C), and Guanine (G) ^5^. Some known polymorphisms are functionally important, being implicated in the origin of disease aggression in the individual. Thus, these substitutions can affect the structure and function of a gene, alter protein synthesis and cell function, and affect AP's progression ^6^. Therefore, individuals with specific types of genotypes may be more susceptible to diseases or may have an increase in their severity ^4^ ^,^ ^5^.

Interleukins 6 and 17 are pro-inflammatory cytokines closely related to osteoclastic processes. Interleukin-6 (IL-6) acts on bone resorption and is responsible for activating osteoclasts, promoting the reabsorption of hard tissues in the presence of infections. It has been suggested as a serious inflammatory cytokine of chronic apical lesions ^2^ ^,^ ^6^ ^,^ ^7^. Interleukin-17 (IL-17) acts in host defense and is involved in inflammatory, autoimmune, metabolic, and neoplastic disorders ^6^ ^,^ ^7^., Although studies have hypothesized over the years that SNPs may alter the production of interleukins in the relationship between microbial factors and heredity, in the presence of AP, ^3^ ^,^ ^7^ ^,^ ^8^ ^,^ ^9^ ^,^ ^10^ the mechanisms, genetic influence, and clinical outcomes remain poorly elucidated ^11^ ^,^ ^12^. In addition, there are no studies that emphasize IL's 6 and 17 and their correlation between AP bone loss and genetic polymorphism, characterizing an important gap in the literature.

Populations may have a high frequency of IL 6 and 17 genetic polymorphisms, which may affect the transcription and expression of these proteins and consequently lead to increased bone loss, represented by large areas of periapical lesions. 

Based on this evidence, this study aimed to verify the frequency of polymorphism in the -174G/C promoter region of the IL-6 gene and the +197 A/G region of the IL-17 gene in patients with asymptomatic periapical inflammatory lesions (APILs) - diagnosed with asymptomatic apical periodontitis (AAP) or chronic apical abscess (CAA) - and verify the existence of correlations between the area of the periapical lesion based on radiographic findings, with age, sex, diagnosis, and presence of the polymorphism, in the evaluated patients.

## Materials and Methods

### Study Typology and Sample Description

Laboratory, observational, descriptive cross-sectional study, submitted and approved by the Research Ethics Committee under number 47921314.6.0000.5207, carried out at the Molecular Biology Laboratory of the Pediatric Onco-Hematology Center, from March 2020 to October 2021, in the State of Pernambuco. The study followed the checklist recommendations of the statement STrengthening the Reporting of Genetic Association Studies STREGA ^13^. 

Samples from a blood bank were used, from patients evaluated, from January 2015 to June 2017, in a Hospital in the state of Pernambuco, attended by spontaneous demand, as they sought the Unit for elective care. This research was based on the study of a population. After screening diagnosed patients and applying the criteria for inclusion of all individuals, the outcome population was chosen.

Patients diagnosed with asymptomatic periapical inflammatory lesions were included. These patients should present one of the clinical-radiographic diagnoses: asymptomatic apical periodontitis (AAP) or chronic apical abscess (CAA). In addition, patients with associated pulpal (pulpitis) and periodontal pathologies, patients with symptomatic periapical pathologies - symptomatic apical periodontitis and acute apical abscess, with endodontic treatment previously performed on the injured tooth, dental wear due to: attrition, abrasion, abfraction or erosion, were excluded.

Information on general health and habits was collected and, according to the physical status classification of the American Society of Anesthesiologists ^15^, only individuals classified as status 1 and 2 were included. Subjects with medical conditions that required the use of systemic bone metabolism modifiers or other drug-assisted therapy (i.e., systemic antibiotics, anti-inflammatories, or hormone therapy) during the previous 6 months were excluded.

Therefore, the population consisted of 492 individuals aged between 18 and 65 years. Clinical information about their general health and habits was collected through anamnesis of all subjects.

Determination of Phenotype

The phenotype (asymptomatic periapical inflammatory lesions) was determined during consultations based on clinical and radiographic aspects.

In this study, we opted for patients with asymptomatic periapical inflammatory lesions, which consist of inflammation and destruction of the apical periodontium, with pulpal origin. In these cases, there is no pain or it is very discreet. As this condition is related to teeth with pulp necrosis, the pulp tests show a negative response, and there is no significant discomfort in response to palpation and percussion. On radiographic examination, apical radiolucency is manifested. Patients with chronic apical abscesses, characterized by an inflammatory process, in which the formation of pus occurs slowly, without significant discomfort for the patient, were also selected. The presence of a fistula is typical of this pathological entity ^14^. 

To avoid distortion, all conventional periapical film radiographs were taken using a bisection angle without a mesial/distal shift. A calibrated and experienced endodontist evaluated each subject's radiographs. In multirooted teeth, the result of the worst root was used to determine the phenotype ^12^. 

All patients presented scores of 4 and 5 according to the periapical index by Orstavik et al. (1986) ^15^, since all of them present periapical lesions. To define the area of the lesions, the radiographs were superimposed on a negatoscope with semi-transparent butter paper, transcribed and interpreted as areas of irregular spaces, and measured with a digital caliper (Absolute Mitutoyo ®). To obtain its values in mm², the largest dimensions were measured, added, and divided by 2, considering, therefore, the area of the periapical lesion. Lesions up to 8.5mm² were classified as small, and larger than 8.5mm², large lesions.

### DNA Extraction and Genotyping

The genetic material from the collected peripheral blood was extracted and quantified using the Salting Out method by Miller et al. (1988) ^16^ and stored in a freezer at -20°C. The amplification reactions had a final volume of 11μL, of which 0.5μL of forward primer (IDT®) (10pmoles), 0.5μL of reverse primer (IDT®) (10pmoles), and 2μL of DNA (50 to 100 ng/μL). Cycling conditions were an initial DNA denaturation at 94°C for 2 minutes, 35 cycles of denaturation at 94°C for 20 seconds, primer annealing at 58°C for 30 seconds, and extension at 72°C for 30 seconds. The final extension was performed at 72ºC for 2 minutes. Genotyping was performed by the polymerase chain reaction-restriction fragment length polymorphism (PCR-RFLP) method. Base pair PCR products and genotyping were analyzed by electrophoresis in a 2% agarose gel and 1X TBE buffer (T base (Tris), Boric acid, and EDTA) stained with ethidium bromide (5 µg ml-1). The primers used for the analyses, restriction enzymes for each SNP, and the characteristics of the genes studied are provided in Box 1.

**Box 1:**
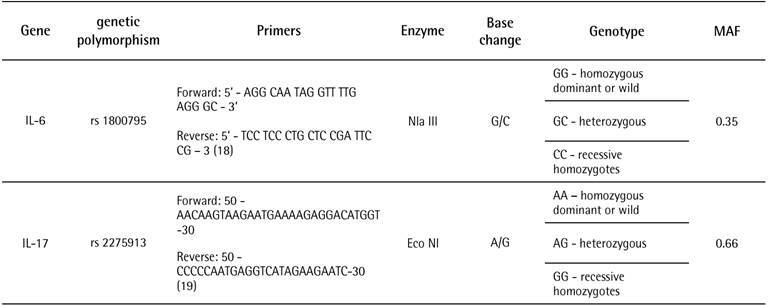
Characteristics of the IL-6 and IL-17 genes studied, markers for analysis, and restriction enzymes for each SNP.

IL-6: interleukin-6; IL-17: interleukin-17;G: Guanine; C: Cytosine; A: Adenine; MAF: Minor allele frequency^20^

### Statistical analysis

For statistical purposes, variables were grouped according to age (18 - 39 years or 40 - 65 years), sex (male or female), and presence or absence of the polymorphism. Small (up to 8.5 mm²) or large (> 8.5 mm²) periapical lesion areas were taken as a determined outcome since all patients in the sample have the phenotype. The database was assembled in Excel 2010 and the statistical analysis was performed in the BioEstat 5.0® Software for Windows.

Fisher's exact and chi-square tests were used to analyze the variable-outcome relationship - area of periapical lesion - with variables and individual characteristics between groups. Fisher's chi-square or exact tests and the odds ratio association measure were used to compare allele and genotype distributions between groups of small and large lesions, in the dominant, heterozygous, and recessive models, and to measure the association between a factor of risk and occurrence of complications. A logistic regression analysis was also performed using gender, age, and the presence of polymorphism as a covariate. To test the relationship between age and lesion area, linear regression was used with the calculation of Pearson's correlation coefficient (r) and determination coefficient (R2). For all analyses, the significance level used was 0.05% (p ≤0.05) and a confidence interval (CI) of 95%. The Hardy-Weinberg equilibrium was assessed using the chi-square test within each polymorphism.

## Results

Of the 492 patients examined, thirty (n=30) had asymptomatic periapical alterations, whereas 83.3% (n=25) had a diagnosis of AAP and 16.7 (n=5) had a chronic apical abscess. Seventy percent of patients (n=21) were male. The smallest lesion area was 4mm² and the largest, 21mm², with a predominance of lesions with an area considered small (≤8.5mm²) (n=53.3%). The most common genotype found for the IL-6 gene was the GC genotype (heterozygous) with 63.4% (n=19) and, for the IL-17 gene, the AG genotype (heterozygous) with 60% (n=18).

Table 1 shows the distribution of frequencies and data from the studied sample. The statistical results showed that there was no statistical significance in the occurrence of periapical lesions correlated with age, gender, and diagnosis, in patients with LIPA's (p ˃0.05). None of the conditions was associated with increased lesion areas (p ˃0.05).

Table 2 shows the distribution of IL-6 and IL-17, genotypes and alleles in individuals with large and small lesions, where the results showed that there was no statistical significance between the distributions of alleles in the polymorphism of IL-6 and IL-17 in the studied groups (p ˃0.05%). Individuals carrying the variant alleles showed no greater or lesser risk in AAP and CAA phenotypes. The same was presented for the genotypes, absence of significant association (p ˃0.05%).

**Figure 1:**
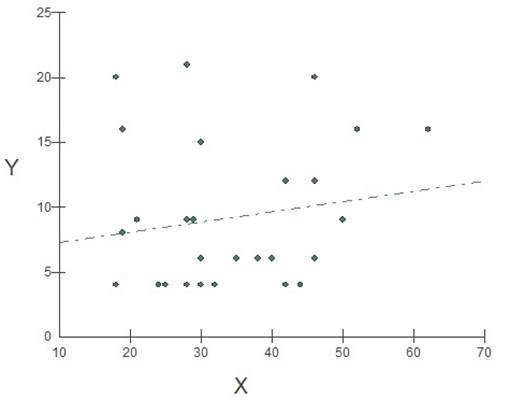
Graphical representation of the linear regression test, in the relationship of X (age in years) and Y (lesion area in mm²), in the studied population, where Y´= a+bX. (a: intercept (6.4362) and b: regression coefficient (0.0802)), p=0.6336, F (regression) r=0.8531 and R2 (determination coefficient) =0.0296, where each point represents a patient. In case of significant association (p ≤0.05%), for every 0.08 years of age, the lesion would increase by 1mm².

In the analysis of the correlation between age and area of the periapical lesion, the results show that there was no significant association (p=0.63) in the relationship between age and increase in the area of the lesion in the studied population. The same applies to the determination coefficient (R2), with no significant association (Figure 1).

**Table 1:** Distribution of frequencies and data on the probability of occurrence of areas of large periapical lesions according to age, gender, and diagnosis, in patients with APILs.

Variables	Large lesion (n=14)	Small lesion (n= 16)	p-value
Age			
18 - 39 n (%)	8	12 (75)	0.25†
40 - 65 n (%)	6 (43)	4 (25)	
Sex			
Male n (%)	11 (79)	10 (62.5)	0.28†
Female n (%)	3 (21)	6 (37.5)	
Diagnosis			
PAA n (%)	12 (85)	13 (81.2)	0.56†
AAC n (%)	2 (15)	3 (18.8)	

† The chi-square test

AAP: Asymptomatic Apical Periodontitis; CAA: Chronic Apical Abscess

**Table 2:** Distribution of IL-6 and IL-17, genotypes and alleles in individuals with large and small lesions.

Gene		Genotype, n (%)				Allele, n (%)			
		CC	GC	GG	p-value*	C	G	p-value*	OR (CI 95%)
IL-6	Large lesions	0 (0)	8 (57.0)	6 (43.0)	0.21	8 (28.5)	20 (71.5)	0.46	0.57 (0.19-1.70)
	Small lesions	1 (6.2)	11 (68.8)	4 (25.0)		13 (40.7)	19 (59.3)		
		AA	AG	GG	p-value*	A	G	p-value*	OR (CI 95%)
IL-17	Large lesions	1 (7.2)	8 (57)	5 (35.7)	0.064	10 (35.7)	18 (64.3)	0.92	1.22 (0.41-5.58)
	Small lesions	0 (0)	10 (62.5)	6 (37.5)		10 (31.3)	22 (68.7)		

IL-6: interleukin-6; IL-17: interleukin-17; CI: confidence interval; OR: odds ratio

*Chi-square test

## Discussion

Studies have sought to explain the relationship between microbial and genetic factors in pulpal and periapical diseases of endodontic origin ^2^ ^,^ ^3^ ^,^ ^6^ ^,^ ^10^ ^,^ ^11^ since they are still scarce. It becomes relevant to know, in individuals, the frequency with which polymorphisms of IL-6 and 17 are present in patients with these pathologies, as they improve the understanding of osteoclastic episodes and bone destruction. ^10^

When we consider the presence of polymorphism for 1 or 2 copies of variant alleles for IL-6 (CC/GC) and IL-17 (GG/AG), both homozygous and heterozygous, our findings identify a high frequency of polymorphism in the studied population. ^10^ These results corroborate the findings of Morsani et al. (2011) ^10^ in which they identified a greater number of polyallelic individuals (homozygous or heterozygous) when compared to wild individuals (without polymorphism) in the study of the pathogenesis of persistent endodontic periapical lesions. In clinical practice, the endodontic treatment performed in these individuals who are predisposed to persistent lesions and/or with large bone losses would be better directed, to mimic the effects of variant alleles, preventing their potentiation and suppressing the harmful effects to the general health of these individuals. ^11^

In the literature, studies reveal an association between IL-6 polymorphism and PA. De Sa et al. (2007) ^22^ identified a significant association between the GG genotype and the G allele of the polymorphism at the -174 (G/C) locus of the IL-6 gene, in women and individuals aged ≤ 35 years, with the presence of symptomatic odontogenic abscess. The GC, CC, and C allele genotypes were also related to a reduced risk of exacerbation, corroborating Fishman et al. (1998) ^23^. These authors state that the G allele is associated with high levels of IL-6 production when compared to the C allele. Considering that IL-6 is an important cytokine of the immune response, the increase in its production can cause great bone destruction.

Already Prso et al. (2007) ^24^ identified elevated levels of IL-6 in symptomatic and asymptomatic lesions. They suggest that the IL-6 gene accompanied by its G allele is related to the increased production of this cytokine in bone loss events. Our results show that there is no association between the presence of IL-6 polymorphism (GC and GG genotypes) and large-diameter lesions (greater bone loss). More studies with asymptomatic lesions, which consider imaging findings, should be carried out so that the role of IL-6 is better related to the size of these lesions, representative of bone loss.

Studies of IL-17 gene polymorphism in patients with LIPA's are scarce. Most of them associate the polymorphism of the IL-17 gene with immunoinflammatory diseases (Sjogren's Syndrome, systemic lupus erythematosus, and rheumatoid arthritis) and periodontal diseases ^25^. Baumeister et al. (2023) ^25^ conducted a study that aimed to test whether IL-17 plays a causal genetic role in the development of periodontitis and identified an overexpression of IL-17, which played a protective role in the initial development of periodontal disease. Our results identified 29 of 30 patients with the G allele (AG and GG), which suggests that this protein may be overexpressed since the G allele is related to the overproduction of IL-17. In this way, it would also promote a protective effect in asymptomatic apical periodontitis and chronic apical abscess.

Studies are needed to elucidate the role of IL-17 in inflammatory periapical diseases of endodontic origin. Identifying the functional role of each polymorphism and its influence on the susceptibility and severity of diseases would not only increase our understanding of the biology of the disease, but would also allow us to elucidate risks, aid in the diagnosis and course of diseases, and determine appropriate treatment regimens to patients ^15^. It is necessary to characterize the populations about the SNPs of the immune system genes related to the inflammatory response. This is important, as it assesses the genetic diversity of the immune system, generating an understanding of how the population responds to inflammatory and/or infectious diseases, allowing tracking of the genetic profile even before the start of treatment.

This study recognizes the sample size as limitation, since they do not express the genetic epidemiological profile and do not represent a population, as it is a sample of spontaneous demand. In Brazil there is high miscegenation, allowing genetic variations in different geographic locations and the miscegenation characteristic of Northeast Brazil. This sample profile has been reported in other studies in the literature ^15^ ^,^ ^25^. Morsani et al. (2011) ^10^ state that the effect of a small sample can generate ambiguous results or an even stronger association. Jakovljevic et al. (2020) ^11^ as well as for our study, the results of high expression of variant alleles, although without significant differences, are possibly due to a small sample. Therefore, new studies with a larger sample number are suggested.

The frequency of homozygous and heterozygous polymorphisms of interleukins 6 and 17 is high in the studied population. The polymorphism of the interleukins studied is not correlated with the increase in the areas of asymptomatic periapical inflammatory lesions.
